# Draft Genome Sequence of “Candidatus Phycosocius bacilliformis,” an Alphaproteobacterial Ectosymbiont of the Hydrocarbon-Producing Green Alga Botryococcus braunii

**DOI:** 10.1128/genomeA.00396-18

**Published:** 2018-05-10

**Authors:** Yuuhiko Tanabe, Haruyo Yamaguchi, Makoto M. Watanabe

**Affiliations:** a Algae Biomass and Energy System R&D Center, University of Tsukuba, Ibaraki, Japan; b Center for Environmental Biology and Ecosystem Studies, National Institute for Environmental Studies, Ibaraki, Japan

## Abstract

“Candidatus Phycosocius bacilliformis” is an alphaproteobacterial ectosymbiont of the hydrocarbon-producing green alga Botryococcus braunii. We sequenced the whole genome of “Ca. P. bacilliformis” BOTRYCO-2, isolated from a two-membered culture with B. braunii. The genome contains approximately 3.3 Mb, with an average G+C content of 56.91% and 3,125 predicted protein-coding genes.

## GENOME ANNOUNCEMENT

The alphaproteobacterium “Candidatus Phycosocius bacilliformis” has been isolated from a nonaxenic strain of the hydrocarbon-producing green alga Botryococcus braunii Ba10 (1). “Ca. P. bacilliformis” is a mutualistic ectosymbiont of B. braunii because it promotes the growth of the alga; efforts to culture this bacterium without B. braunii have been unsuccessful (1). A previous study reported that bacteria closely related to “Ca. P. bacilliformis” genetically are frequently found in freshwater cyanobacterial blooms (1). This further suggests that “Ca. P. bacilliformis,” and species related to it, are associated with phytoplankton in addition to the original source alga B. braunii. However, the molecular mechanism underlying this symbiotic relationship is enigmatic. To obtain an insight into the molecular basis of this algal-bacterial symbiotic relationship, we sequenced the whole genome of “Ca. P. bacilliformis” BOTRYCO-2, which is the only available strain of this bacterium.

“Ca. P. bacilliformis” BOTRYCO-2 cells were collected from a 60-ml two-membered culture comprising “Ca. P. bacilliformis” BOTRYCO-2 and B. braunii Ba10 (1). Genomic DNA was extracted using NucleoBond AXG columns with a buffer set III (Macherey-Nagel, Düren, Germany). DNA was fragmented to approximately 550 bp using the Covaris M220 instrument (Covaris, Woburn, MA). A 550-bp fragmented library was constructed using the NEBNext Ultra DNA library prep kit for Illumina (New England Biolabs, Ipswich, MA). The DNA was sequenced using the MiSeq platform (Illumina, San Diego, CA, USA) with the 600-cycle MiSeq V3 reagent kit. This sequencing resulted in 1,006,529 paired-end reads. Low-quality reads/bases were filtered using Trimmomatic version 0.36 (2), and *de novo* assembly was performed using SPAdes version 3.10.1 (3). The resulting genome comprised 21 contigs of 3,332,539 bp. The average genome coverage of the paired-end reads was 121×. The maximum contig length was 701,368 bp. The draft genome of “Ca. P. bacilliformis” BOTRYCO-2 was annotated using Prokka version 1.12 (4). The genome comprised 3,125 predicted protein-coding sequences (CDSs), including 1,250 hypothetical proteins and 45 RNAs. The G+C content of the genome was 56.91%. Bacterial symbionts are known to provide algal hosts with essential vitamins, including thiamine, biotin, and cobalamin, which some algae cannot synthesize *de novo* (5). None of the genes required for the synthesis of these three vitamins were detected in the genome, excluding the possibility that “Ca. P. bacilliformis” can promote the growth of its host alga by supplying vitamins. Unexpectedly, the complete set of genes synthesizing bacteriochlorophyll *a*, as well as those synthesizing carotenoid spirilloxanthin (6), were detected, suggesting that “Ca. P. bacilliformis” is a photosynthetic bacterium. This draft genome sequence can provide genetic information for the future characterization of the molecular basis of algal-bacterial symbiosis between “Ca. P. bacilliformis” BOTRYCO-2 and its host green alga.

### Accession number(s).

This whole-genome shotgun project has been deposited in DDBJ/GenBank under the accession no. BFBR01000001 to BFBR01000021.
